# Leaf transcriptome profiling of contrasting sugarcane genotypes for drought tolerance under field conditions

**DOI:** 10.1038/s41598-022-13158-5

**Published:** 2022-06-01

**Authors:** Danyel Fernandes Contiliani, João Felipe Carlos de Oliveira Nebó, Rafael Vasconcelos Ribeiro, Larissa Mara Andrade, Rafael Fávero Peixoto Júnior, Carolina Gimiliani Lembke, Ricardo Silverio Machado, Daniel Nunes Silva, Mariana Belloti, Gláucia Mendes de Souza, Dilermando Perecin, Tiago Campos Pereira, Regina Célia de Matos Pires, Patrícia Rezende Fontoura, Marcos G. A. Landell, Antonio Figueira, Silvana Creste

**Affiliations:** 1 Instituto Agronômico (IAC), Centro de Cana, Ribeirão Preto, Brazil; 2 Programa de Pós-Graduação em Genética, Faculdade de Medicina de Ribeirão Preto, Universidade de São Paulo, Ribeirão Preto, Brazil; 3 Centro de Energia Nuclear na Agricultura (CENA), Universidade de São Paulo, Piracicaba, Brazil; 4 Laboratory of Crop Physiology, Department of Plant Biology, Institute of Biology, University of Campinas, Campinas, Brazil; 5 Departamento de Bioquímica, Instituto de Química, Universidade de São Paulo, São Paulo, Brazil; 6 Instituto Agronômico (IAC), Centro de Ecofisiologia e Biofísica, Campinas, Brazil; 7 Faculdade de Ciências Agrárias e Veterinárias, Universidade Estadual Paulista Júlio de Mesquita Filho, Jaboticabal, Brazil; 8 Departamento de Biologia, Faculdade de Filosofia, Ciências e Letras de Ribeirão Preto, Universidade de São Paulo, São Paulo, Brazil; 9 Jalles Machado Sugar Mill, Goianesia, Brazil

**Keywords:** Transcriptomics, Plant molecular biology

## Abstract

Drought is the most detrimental abiotic stress to sugarcane production. Nevertheless, transcriptomic analyses remain scarce for field-grown plants. Here we performed comparative transcriptional profiling of two contrasting sugarcane genotypes, ‘IACSP97-7065’ (drought-sensitive) and ‘IACSP94-2094’ (drought-tolerant) grown in a drought-prone environment. Physiological parameters and expression profiles were analyzed at 42 (May) and 117 (August) days after the last rainfall. The first sampling was done under mild drought (soil water potential of −60 kPa), while the second one was under severe drought (soil water potential of −75 kPa). Microarray analysis revealed a total of 622 differentially expressed genes in both sugarcane genotypes under mild and severe drought stress, uncovering about 250 exclusive transcripts to ‘IACSP94-2094’ involved in oxidoreductase activity, transcriptional regulation, metabolism of amino acids, and translation. Interestingly, the enhanced antioxidant system of ‘IACSP94-2094’ may protect photosystem II from oxidative damage, which partially ensures stable photochemical activity even after 117 days of water shortage. Moreover, the tolerant genotype shows a more extensive set of responsive transcription factors, promoting the fine-tuning of drought-related molecular pathways. These results help elucidate the intrinsic molecular mechanisms of a drought-tolerant sugarcane genotype to cope with ever-changing environments, including prolonged water deficit, and may be useful for plant breeding programs.

## Introduction

Sugarcane (*Saccharum* spp.) is a C4 grass crop widely cultivated in tropical and subtropical regions, as it represents the main source of feedstock for sugar and bioethanol production. The global harvest area encompasses approximately 26 million hectares^1^, and Brazil is the leading producer responsible for about 35% of world production^2^. However, sugarcane production has been affected by unfavorable climatic conditions, which are increasing in frequency and intensity. Drought is considered the most limiting abiotic stress to sugarcane cultivation, impairing yield even during the rainy season due to dry summer spells^3^. For instance, water scarcity was responsible for progressive losses in sugarcane yield in the last crop seasons, leading to an estimated decrease of 15% in the Southeastern Brazilian region in 2021^2^. Therefore, as climate change intensifies, sugarcane crops are harmed; hence the sugar and bioenergy industries strive for sustainable cultivars.

Water stress triggers several morphological and physiological changes in sugarcane plants, and its impact on plant development varies according to the genotype, plant organ, drought intensity, and exposure^4^. The most common drought-induced plant responses are stomatal closure and impairment of cell division and photosynthesis^4,5^. Moreover, plant defense also relies on abscisic acid (ABA)-dependent and -independent molecular responses involving cell signaling and regulation of drought-induced gene expression^6^. In particular, sugarcane cultivars exhibit a differential response to water stress^7–10^, among which a tolerant genotype (IACSP94-2094) displays an enhanced antioxidant system that promotes the recovery of photosynthesis after drought stress and supports plant growth^7^. Thus, screening for drought-tolerant and high-yielding genotypes^8^ has become a priority in sugarcane breeding programs.

Plant adaptation to drought stress is tightly controlled by a coordinated set of signaling networks, which switch on/off clusters of genes that directly affect the plant response^9^. Drought-associated gene expression changes were thoroughly investigated in leaves of several plant species, including sugarcane^10–17^. These transcriptomic profiling-based studies depicted a large number of genes that are differentially expressed in water-limited scenarios, which are involved in osmotic and oxidative stress protection, secondary metabolite biosynthesis, photosynthesis, transcriptional regulation, among others^10–17^. Some of these studies have employed comparative analyses between transcriptional profiles of sugarcane genotypes that exhibit contrasting responses to drought (i.e., tolerant versus sensitive genotypes)^11,13,15,17,18^. Notably, the genotype-comparative approach has gained prominence because of its power to discern the particular molecular elements of tolerant genotypes.

Although the transcriptional dissection of plants subjected to field conditions may provide fundamental insights into the stress-dependent molecular networks, most sugarcane transcriptional profiling studies addressing drought tolerance were conducted under greenhouse conditions^11,13,17,18^; except for Belesini et al.^15^, which performed de novo transcriptome assembly of sugarcane leaves grown under field conditions, but without experimental validation of gene expression data. In fact, large-scale experiments under ever-changing environmental settings are costly and labor-intensive and require an integrated effort of players from different sectors. Still, this approach may promote the discovery of new biotechnological leads for agronomic traits.

Efforts for long-term field experimentation, rather than short-term greenhouse drought simulations, are needed to address the challenges of drought in agriculture, in which water restriction persists for several months. In this regard, we performed microarray analysis to characterize transcriptional profiles in leaves of two sugarcane genotypes, ‘IACSP97-7065’ (drought-sensitive) and ‘IACSP94-2094’ (drought-tolerant), at the grand growth stage, also known as the formative phase. Plants were cultivated in natural drought-prone soil to ensure the fine-tuned and coordinated physiological and molecular responses to an environmental scenario. Differentially expressed genes (DEGs) were investigated at 42 (May) and 117 (August) days after the last rainfall, when plants were facing mild (soil water potential of −60 kPa) and severe (soil water potential of −75 kPa) drought stress, respectively. The sugarcane genotypes were compared to elucidate the critical biological processes that underpin drought tolerance. Furthermore, microarray expression data were validated by quantitative real-time PCR (RT-qPCR).

## Results

### Physiological responses to drought

**Figure 1:**
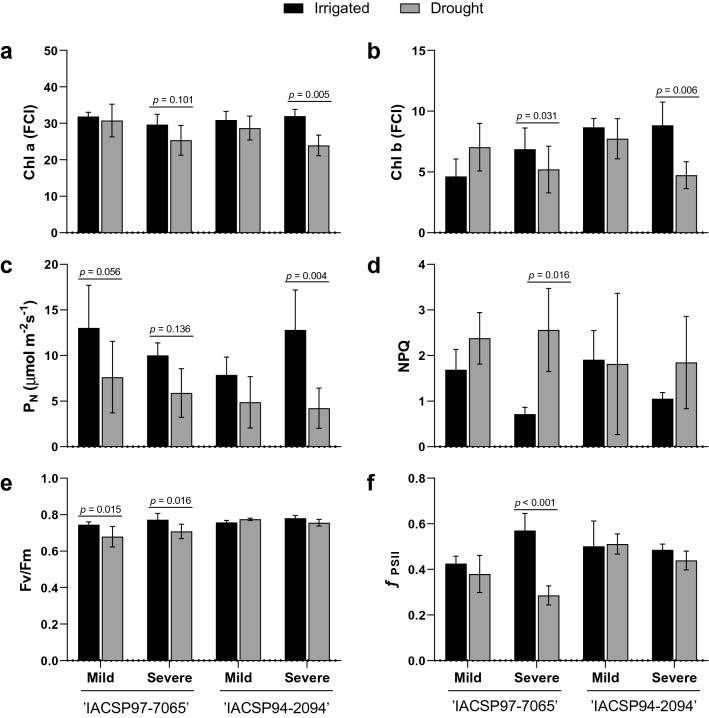
Physiological parameters of sugarcane leaves under drought conditions. Drought-sensitive (IACSP97-7065) and -tolerant (IACSP94-2094) sugarcane genotypes were evaluated at 42 (mild drought) and 117 (severe drought) days after the last rainfall and compared with the corresponding controls (irrigated). From the 42nd day after the last rainfall, the soil water potential has progressively reduced from −60 to −75 kPa. (**a**) Chlorophyll a (Chl a), (**b**) Chlorophyll b (Chl b), (**c**) Leaf CO_2_ assimilation (*P*_*N*_), (**d**) Non-photochemical quenching (*NPQ*) processes, (**e**) Potential quantum efficiency of PSII (*F*_v_*/F*_m_), (**f**) Effective quantum efficiency of PSII (Φ_PSII_). Only statistical differences between irrigated vs. drought within the same genotype are shown.

The impacts of drought stress on sugarcane plants were investigated by analyzing physiological parameters. As ‘IACSP97-7065’ presented decreases in CO_2_ assimilation rate (41%) and photochemical efficiency (8%) after 42 days of the last rainfall (Fig. 1c,e), it can be argued that this genotype was facing mild drought stress. On the other hand, ‘IACSP94-2094’ did not show any changes in the same parameters analyzed at the same sampling point. After 117 days after the last rainfall, both ‘IACSP97-7065’ and ‘IACSP94-2094’ genotypes showed reductions in total chlorophyll content (16% and 30%, respectively) and CO_2_ assimilation (41% and 67%, respectively) (Fig. 1a–c), indicating a severe drought stress condition. However, only ‘IACSP97-7065’ exhibited an increase in non-photochemical quenching processes (260%), and decreases in potential (8%) and effective (50%) quantum efficiencies of PSII (Fig. 1d–f).

### Differential gene expression analyses

**Figure 2:**
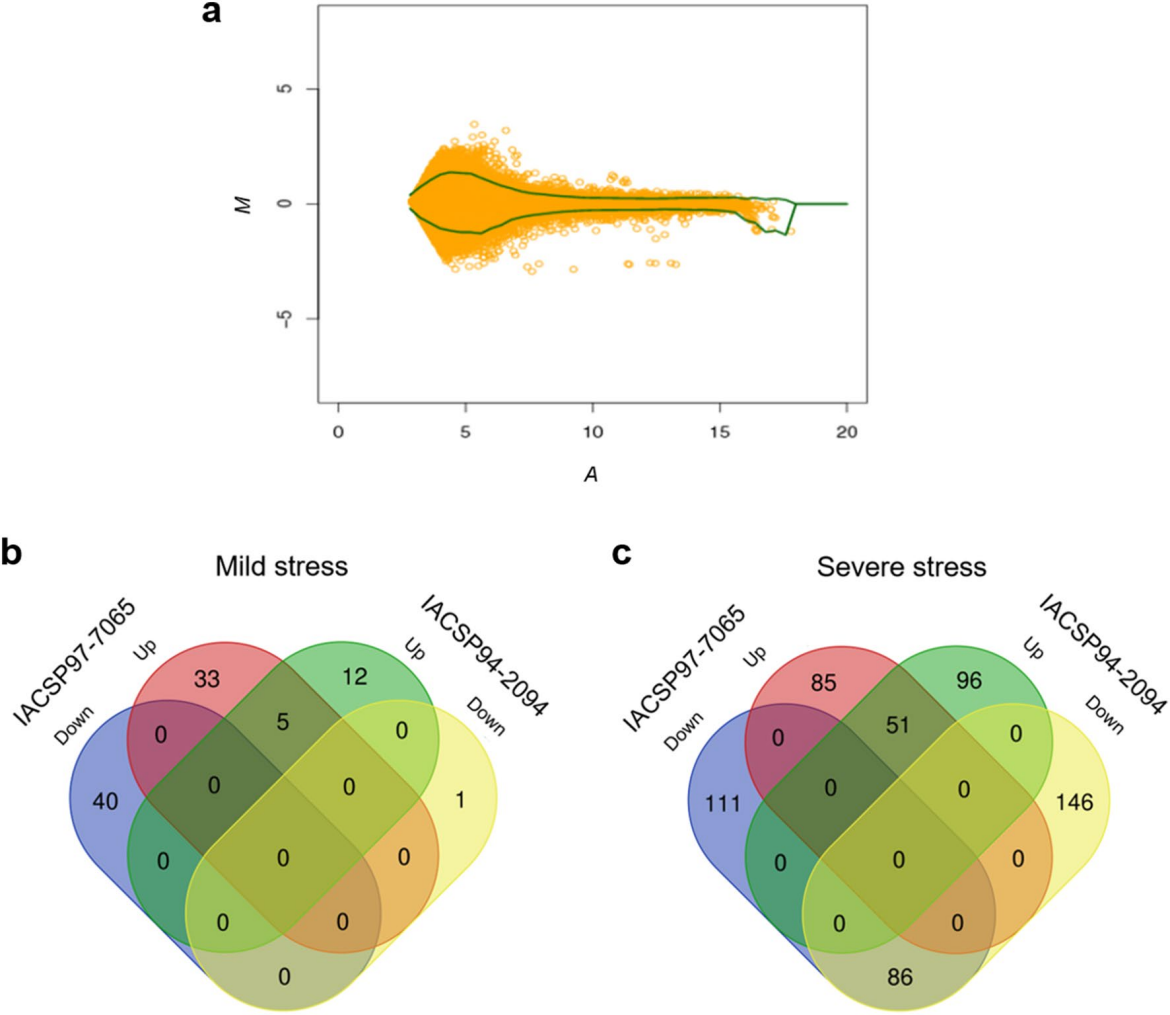
Differentially expressed genes in leaves of contrasting sugarcane genotypes. (**a**) MA-plot of the microarray experiment. The spots located outside the green line (confidence level set to *p* = 0.9) indicate differentially expressed genes (DEGs). Venn diagrams represent the DEGs identified as down- and up-regulated in leaves of sugarcane sensitive (IACSP97-7065) and tolerant (IACSP94-2094) genotypes under (**b**) mild and (**c**) severe drought stress.

Expression profile analysis of 14,552 Sugarcane Assembled Sequences (SAS) (Fig. 2a) revealed a set of 622 differentially expressed genes (DEGs) (4.3%) between sugarcane genotypes, ‘IACSP97-7065’ and ‘IACSP94-2094’, under drought conditions (see Supplementary Table S1 online). Under mild drought stress, ‘IACSP97-7065’ and ‘IACSP94-2094’ displayed 73 (40 down- and 33 up-regulated) and 13 (1 down- and 12 up-regulated) exclusive DEGs, respectively, as compared to their respective controls (Fig. 2b). Among these, only five DEGs were found to be commonly up-regulated in both genotypes. Under severe drought stress (Fig. 2c), 196 and 242 DEGs were exclusively found in ‘IACSP97-7065’ (111 down- and 85 up-regulated) and ‘IACSP94-2094’ (146 down- and 96 up-regulated) genotypes, respectively, compared to their respective controls. Among these, 137 DEGs (86 down- and 51 up-regulated) were shared between both genotypes.

### Functional annotation of differentially expressed genes

All DEGs were searched against the vascular plant sequences in NCBI non-redundant (nr) database (see Supplementary Table S2 online). Annotation of down- and up-regulated DEGs in mild drought stress revealed that 67 (86%) and 18 (100%) showed similarity to functional proteins in ‘IACSP97-7065’ and ‘IACSP94-2094’ genotypes, respectively. Moreover, the drought sensitive genotype ‘IACSP97-7065’ exhibited four DEGs (5%) that show similarity to hypothetical proteins. Under severe drought stress, ‘IACSP97-7065’ and ‘IACSP94-2094’ genotypes displayed 323 and 370 DEGs with matches in the NCBI database, respectively; among which, 296 (91.6%) and 340 (91.9%) were similar to functional proteins, and 5% of the DEGs showed similarity to hypothetical proteins in both genotypes.

**Figure 3:**
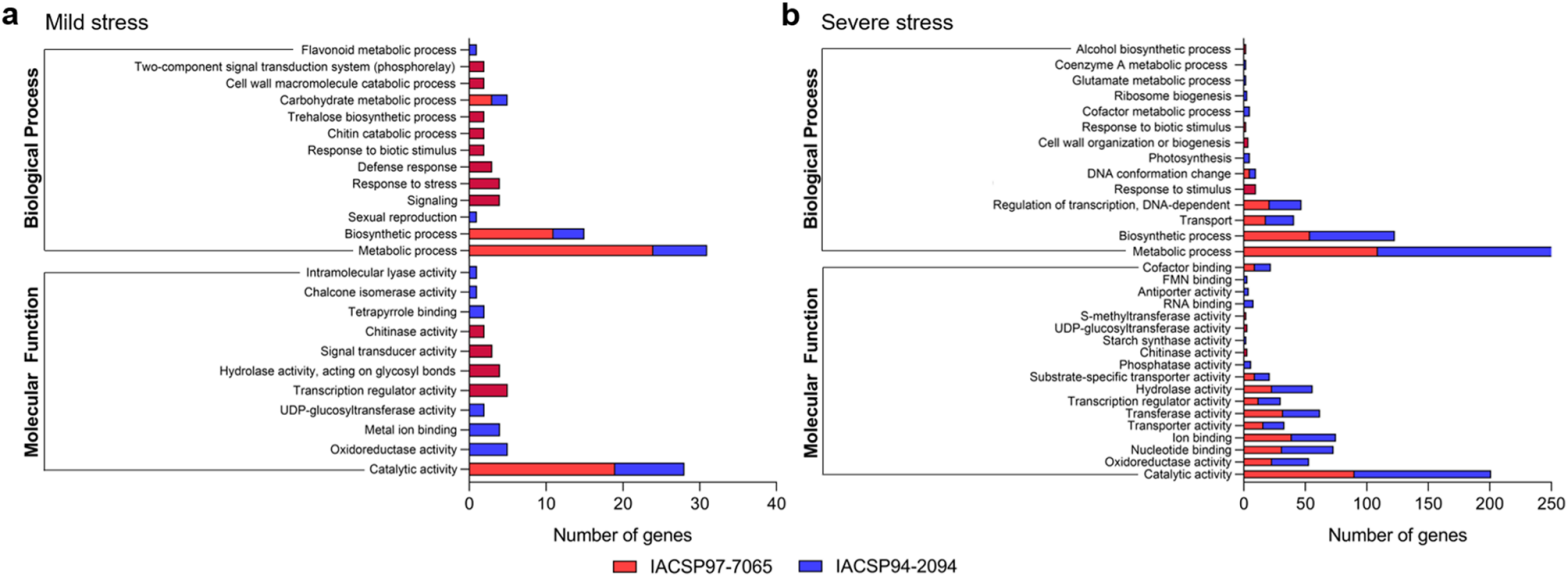
Functional enrichment of differentially expressed genes. Statistically significant enriched GO (Gene Ontology) terms encompassing ‘biological process’ and ‘molecular function’ are represented for the sensitive (IACSP97-7065—red) and tolerant (IACSP94-2094—blue) sugarcane genotypes under (**a**) mild and (**b**) severe drought stress (*p* ≤ 0.05).

Functional enrichment analysis of the DEGs was performed for both genotypes under mild and severe drought stress (Fig. 3a,b). Under mild stress, only seven and 38 DEGs were associated with significantly enriched GO terms in ‘IACSP97-7065’ and ‘IACSP94-2094’ genotypes, respectively (*p-*values ranging from 6.8 × 10^−7^ to 0.048). Only the enriched GO terms “catalytic activity” (GO:0003824), “metabolic process” (GO:0008152), “biosynthetic process” (GO:0009058), and “carbohydrate metabolic process” (GO:0005975) were common between both genotypes. Thus, genotype-specific biological activities on the onset of drought stress could be identified such as “oxidoreductase activity” (GO:0016491), “metal ion binding” (GO:0046872), “UDP-glucosyltransferase activity” (GO:0035251), “transcriptional regulator activity” (GO:0140110), “hydrolase activity” (GO:0016787), “signal transducer activity” (GO:0004871), “chitinase activity” (GO:0004568), “tetrapyrrole binding” (GO:0046906), “chalcone isomerase activity” (GO:0045430), “intramolecular lyase activity” (GO:0016872), “sexual reproduction” (GO:0019953), “signaling” (GO:0023052), “response to stress” (GO:0006950), “defense response” (GO:0002217), “response to biotic stimulus” (GO:0009607), “chitin catabolic process” (GO:0006032), “trehalose biosynthetic process” (GO:0005992), “cell wall macromolecule catabolic process” (GO:0044039), “two-component signal transduction system—phosphorelay” (GO:0070297), and “flavonoid metabolic process” (GO:0009812). Moreover, severe drought caused a higher number of DEGs associated with significantly enriched GO terms in ‘IACSP97-7065’ (196) and ‘IACSP94-2094’ (237) genotypes (*p-*value ranging from 4 × 10^−18^ to 0.048). Most of the enriched terms are common between the genotypes, suggesting their conserved biological activities under severe drought, such as “catalytic activity” (GO:0003824), “oxidoreductase activity” (GO:0016491), “nucleotide binding” (GO:0000166), “ion binding” (GO:0043167), “transporter activity” (GO:0005478), “transcription regulator activity” (GO:0030528), “hydrolase activity” (GO:0016787), “substrate-specific transporter activity” (GO:0005386), “cofactor binding” (GO:0048037), “transport” (GO:0006810), “transferase activity” (GO:0016740), “biosynthetic process” (GO:0044711), “metabolic process” (GO:0044710), “regulation of transcription, DNA-dependent” (GO:0043193), and “DNA conformation change” (GO:0071103). However, some particular enriched GO terms were also found. While “UDP-glucosyltransferase activity” (GO:0035251) and “response to stimulus” (GO:0050896) were significant only in ‘IACSP97-7065’ genotype, “photosynthesis” (GO:0015979) and “antiporter activity” (GO:0015297) were significant only in ‘IACSP94-2094’.

### Analysis of deregulated pathways under drought

Differentially expressed genes in ‘IACSP97-7065’ and ‘IACSP94-2094’ were classified according to KEGG pathway categories (*p* ≤ 0.05) for mild and severe drought stresses (Table 1). Under mild drought stress, ‘IACSP97-7065’ displayed DEGs associated with metabolism of energy, amino acids, carbohydrates, lipids, and environmental adaptation; conversely, the drought-tolerant genotype ‘IACSP94-2094’ showed no enriched pathways. Moreover, KEGG pathway categories were substantially enriched for both genotypes under severe stress. Besides the induced pathways due to mild drought stress, both genotypes also showed activities associated with the metabolism of other amino acids, and translation and transcription pathways. Regarding the common plant slim categories between both genotypes, enriched DEGs in ‘IACSP94-2094’ genotype often outnumbers those of ‘IACSP97-7065’.

**Table 1:**
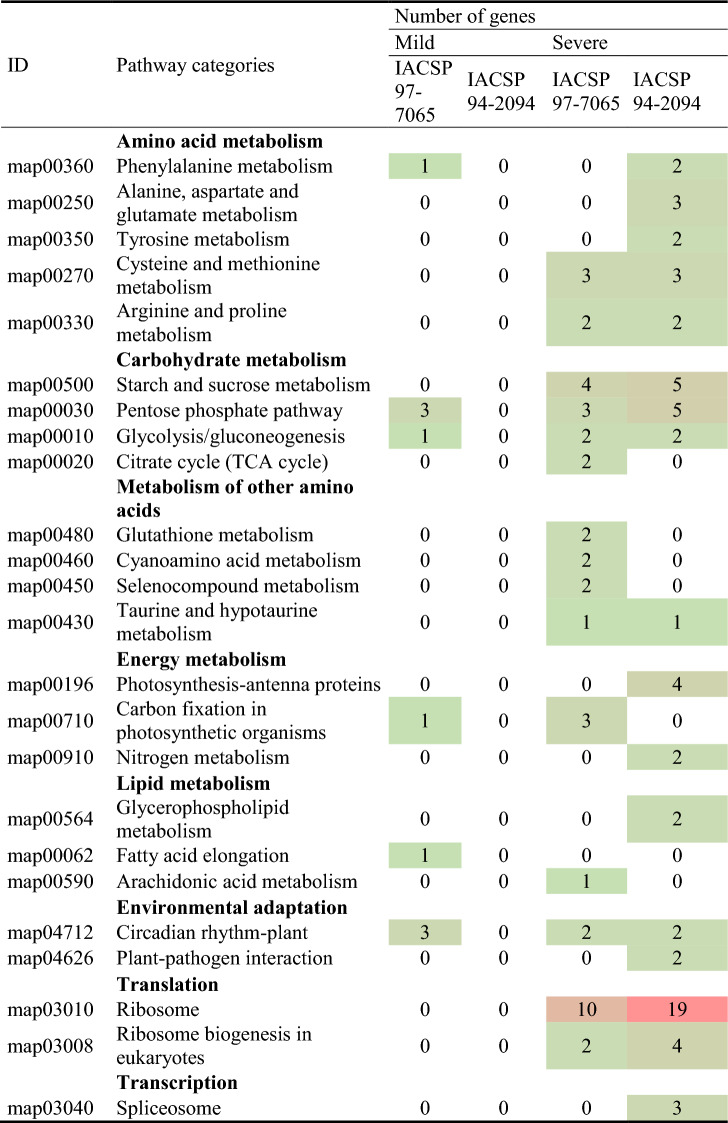
KEGG pathway categories.

Significant enriched KEGG categories represent differentially expressed genes of ‘IACSP97-7065’ and ‘IACSP94-2094’ genotypes under mild and severe drought stress (*p* ≤ 0.05).

Noteworthy, genotype-specific plant slim categories were also revealed. DEGs of the drought-sensitive genotype ‘IACSP97-7065’ were classified into exclusive categories, such as citrate cycle, fructose and mannose metabolism, pyruvate metabolism, glyoxylate and dicarboxylate metabolism, amino sugar and nucleotide sugar metabolism, glutathione metabolism, cyanoamino acid metabolism, selenocompound metabolism, carbon fixation in photosynthetic organisms, fatty acid elongation, and arachidonic acid metabolism. On the other hand, the drought-tolerant genotype ‘IACSP94-2094’ comprises DEGs that are represented by photosynthesis (antenna proteins), plant-pathogen interaction, spliceosome and metabolism of amino acids (i.e., alanine, aspartate, glutamate, tyrosine), glycerophospholipid, and nitrogen. The identification of genes involved in each enriched KEGG pathway category is shown in Supplementary Table S3 online.

### Validation of microarray results

**Figure 4:**
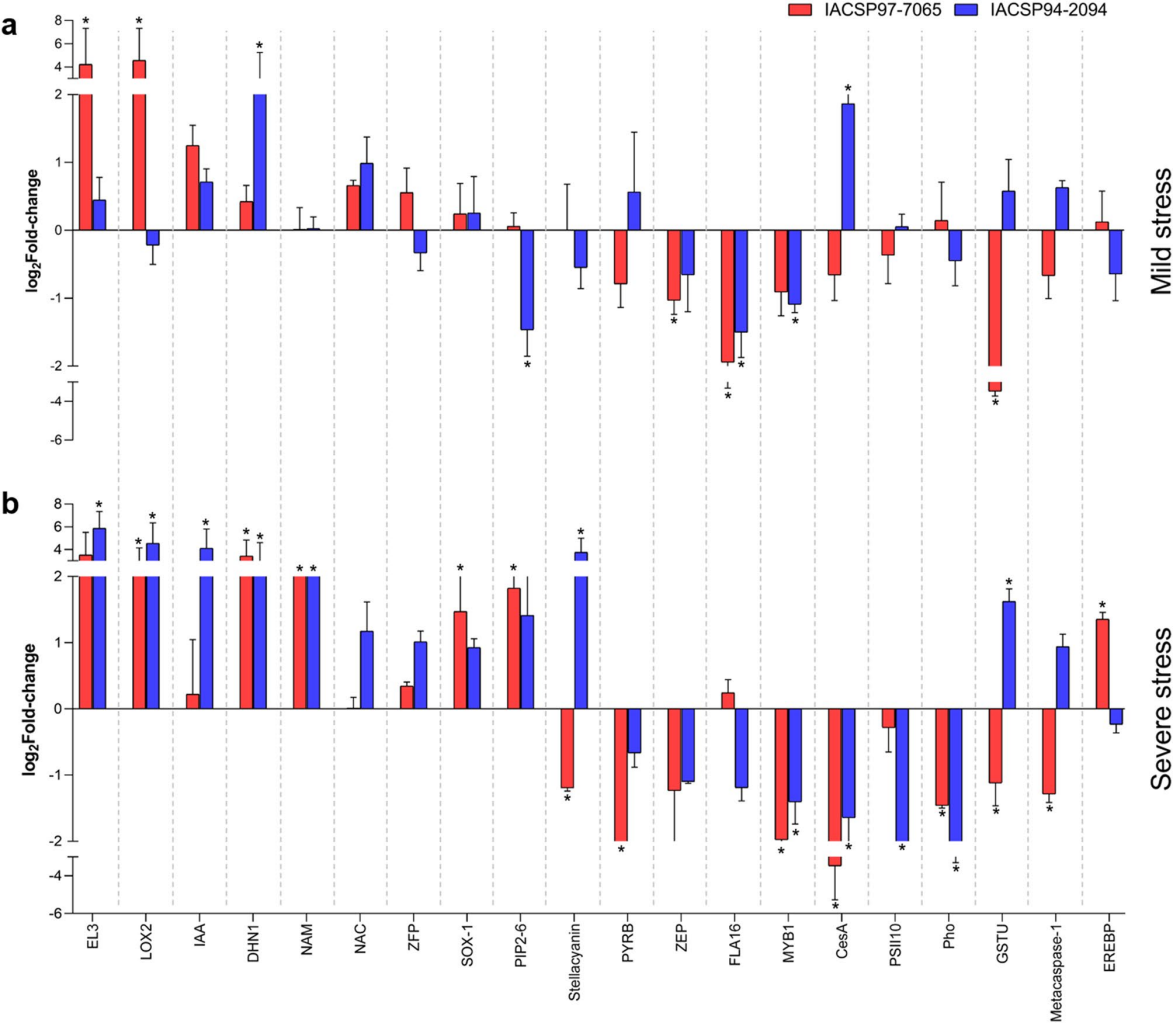
Gene expression validation by quantitative real-time PCR (RT-qPCR) analysis. Leaf transcriptional changes are represented as log_2_Fold-change (bars) for both sensitive (IACSP97-7065—red) and tolerant (IACSP94-2094—blue) sugarcane genotypes under (**a**) mild and (**b**) severe drought stress, compared to the respective irrigated controls. Gene symbol was based on orthology to rice (*Oryza sativa*). Error bar represents the standard deviation and the asterisk (*) indicates statistical significance (*p* ≤ 0.05).

The correlation analysis of DEGs detected by microarray and validated by RT-qPCR indicated a strong similarity (Pearson’s *r* = 0.849, *p-*value = 0.001; Spearman’s rank = 0.834; *p-value* < 0.0001) for the gene expression data (see Supplementary Fig. S1 online), which supports the reproducibility and accuracy of the transcriptional profiles presented in this study. As shown in Fig. 4, RT-qPCR analysis confirmed the differential expression of 18 genes in at least one of the sugarcane genotypes. In common, only the FLA16 gene was found differentially expressed for both genotypes under mild stress. Moreover, six genes were significantly differentially expressed in both genotypes under severe stress, among which LOX2, DHN1, and NAM were up-regulated, whereas MYB1, CesA, and PHO1 were down-regulated.

Remarkably, most validated gene expressions were found to be genotype-specific, some with contrasting expression profiles between ‘IACSP97-7065’ and ‘IACSP94-2094’. In the mild stress condition (Fig. 4a), the drought-sensitive genotype, ‘IACSP97-7065’, showed two up-regulated (EL3 and LOX2) and one down-regulated (ZFP) gene expression. Conversely, the tolerant genotype, ‘IACSP94-2094’, displayed two up-regulated (DHN1, CesA) and two down-regulated gene expressions (PIP2-6 and MYB1). During severe stress (Fig. 4b), the drought-sensitive genotype showed three up-regulated (SOX-1, PIP2-6, and EREBP) and four down-regulated gene expressions (Stellacyanin, PYRB, GSTU, and Metacaspase-1). In turn, the drought-tolerant genotype presented four up-regulated (EL3, IAA, Stellacyanin, and GSTU) and only one down-regulated (PSII10) gene expression. Interestingly, Stellacyanin and GSTU gene expressions were found with contrasting gene expression profiles between the genotypes. The SAS identification of each gene analyzed by RT-qPCR is provided in Supplementary Table S4 online.

## Discussion

Water is essential for plant development, and the sugarcane formative phase (*i.e.,* grand growth stage) is critically dependent on water supply^19^ since the shortage can reduce cane yield by up to 70%^20^. In a drought scenario, physiological, biochemical, and molecular responses are triggered specifically to the organ type^21^. In general, low water availability is detrimental to photosynthesis, leading to low functioning of photosystem II (PSII)^22,23^, which turns the maximum quantum efficiency of PSII (*F*_v_/*F*_m_) into an indicator of drought stress^24^. Moreover, in the water-limiting context, the absorbed light energy exceeds its use in photosynthetic reactions, which results in energy dissipation by non-photochemical processes^25^. Accordingly, physiological analyses in leaves of drought-stressed sugarcane cultivars (‘IACSP97-7065’ and ‘IACSP94-2094’) revealed several changes in photosynthetic variables (Fig. 1), with long term exposure to low water availability intensifying plant responses. However, only ‘IACSP97-7065’ was promptly affected at 42 days after the last rainfall and showed impacts on carbon assimilation and *F*_*v*_*/F*_*m*_, indicating mild drought stress. In addition, this sensitive genotype showed increases in the non-photochemical quenching and reduced potential and effective quantum efficiencies of PSII at 117 days after the last rain (Fig. 1d–f), which revealed plants were facing severe drought stress. These results suggest that excessive light energy was partially dissipated by non-photochemical processes (*NPQ*) and caused damage to PSII. On the other hand, ‘IACSP94-2094’ showed no decreases in *F*_v_/*F*_m_ and Φ_PSII_, indicating that the maximum conversion of light energy into chemical energy was not affected by drought stress. Interestingly, such photosynthetic response of ‘IACSP94-2094’ to water deficit was previously highlighted under greenhouse conditions^26,27^ and confirmed its drought tolerance when cultivated in a drought-prone field. Curiously, the ‘photosynthesis—antenna proteins’ pathway was enriched only for ‘IACSP94-2094’ under severe drought (Table 1), representing the repressions of light-harvesting chlorophyll-binding proteins encoding genes. We would argue that this may be a molecular strategy to avoid excessive energetic pressure in PSII^28^ and partially justify the maintenance of *F*_v_/*F*_m_ and Φ_PSII_ even after 117 days of water deficit.

Transcriptional responses comprise multiple sets of drought-responsive genes that are spatiotemporally repressed or induced in an intricate manner^9^. Global gene expression regulation has already been investigated in sugarcane plants under drought conditions^10–18^. However, few studies have addressed the contrasting transcriptional profile between drought-tolerant and drought-sensitive sugarcane genotypes^11,13,15,17,18^; even rarer are those conducted within natural settings, exploring the dynamic influence of the environment^15^. In this study, we used microarray technology to decipher the distinct molecular adaptations of contrasting sugarcane (*Saccharum* spp.) genotypes, ‘IACSP97-7065’ (drought-sensitive) and ‘IACSP94-2094’ (drought-tolerant), to long-term water-deficit stress under natural environmental conditions. From the analysis of 14,552 SAS, we identified a set of 662 (4.6%) DEGs in sugarcane leaves under mild and severe stress (Fig. 2a). Among those, most are not shared between the sugarcane genotypes, which reinforces their divergent responses to drought at the transcriptional level (Fig. 2b,c). Yet, several DEGs found in both sugarcane genotypes are similar to the major drought stress-related hub genes (DSRhub genes) from sorghum (*Sorghum bicolor*) regulatory networks^29^, including transcription factors and other regulatory proteins. In particular, the lower number of DEGs in the ‘IACSP94-2094’ genotype under mild stress (fivefold less than ‘IACSP97-7065’) may be attributed to its drought-tolerant profile, in which a mild drought is unlikely to cause cell damage and/or the activation of drought-responsive signaling networks. Moreover, the functional enrichment analyses revealed that the DEGs of the sensitive and tolerant sugarcane genotypes are associated with different cellular processes (Fig. 3). Although hormone signaling pathways were not significantly enriched, some related genes already reported in drought-stressed sugarcane^30^ and sorghum^29^ were genotype-specific regulated. For instance, whereas only ‘IACSP97-7065’ shows the ethylene biosynthesis pathway induction, our RT-qPCR analysis revealed the auxin-responsive protein (IAA12–SCJFRT1007H07.g) as up-regulated in only ‘IACSP94-2094’ under severe drought stress (Fig. 4b). Collectively, the microarray data from this study may indicate distinct strategies of those plants in coping with drought.

Environmental stresses such as drought are conservatively sensed by plants, leading to the overproduction of reactive oxygen species (ROS) as key components of signal transduction pathways; however, with detrimental effects to cells^31^. The oxidative stress in sugarcane causes significant damage to PSII and can virtually abolish photosynthetic efficiency and electron transport rate^32^. Under mild stress, despite the lower number of DEGs in the ‘IACSP94-2094’ genotype, some were uniquely associated with redox enriched pathways. Those included polyphenol oxidase (SCSGLR1045A10.g), cinnamyl alcohol dehydrogenase (SCSBAD1054A07.g), chalcone-flavanone isomerase 1 (SCCCLR1C03G09.g), and cytochrome P450-related genes (SCCCCL3001H12.g), which have been reported to participate in acclimation to water and oxidative stresses in sugarcane^33–35^, sorghum^29^ and model species^36^. In special, induction of CYP450 gene expression may represent an early mechanism for the tolerant genotype to avoid oxidative injury induced by high levels of hydrogen peroxide, as reported in drought-stressed CYP450-RNAi knockdown cotton plants^37^. Remarkably at severe drought stress, the tolerant genotype showed a greater number of deregulated genes in oxidoreductase activities than the sensitive genotype, with 20 exclusive DEGs. Additionally, some common oxidoreductase-related DEGs may exhibit relevant opposite regulations on gene expression of sugarcane genotypes. For example, glutathione s-transferase (GSTU) is strongly associated with preventing oxidative burst in plant cells exposed to abiotic stress^38^. Interestingly, our RT-qPCR results confirmed that GSTU was down-regulated in the sensitive genotype (log_2_FC = −1.12) and up-regulated in the tolerant genotype (log_2_FC = 1.62) under severe drought stress. In a greenhouse study, ‘IACSP94-2094’ exhibited the antioxidant system as a key feature for the recovery of the photosynthetic system after exposure to drought stress, thus enabling normal plant growth^7^. Overall, these results reinforce that the tolerant genotype ‘IACSP94-2094’ harbors an enhanced antioxidant system that might alleviate drought-induced damage.

Drought tolerance in plants is ensured by signaling pathways that elicit critical players in gene expression modulation—the transcription factors (TFs)^39^. Despite the lack of enriched transcriptional regulators in the ‘IACSP94-2094’ genotype under mild stress, we identified that R2R3-MYB was down-regulated by RT-qPCR analysis. We previously reported that overexpression of the most abundant alternative form of ScMYBAS1 transcript (ScMYBAS1-2) resulted in reduced biomass in transgenic rice plants subjected to drought^40^. Accordingly, the repression of R2R3-MYB was also detected in another tolerant sugarcane genotype (KPS01-12) under drought stress^17^, suggesting this down-regulation as a beneficial trait when plants face drought. Under severe drought stress, the enriched TF genes in ‘IACSP94-2094’ outnumbered those of the sensitive genotype, unveiling particular up- (*MADS-box 1*, SCCCCL4015C03.g; auxin-responsive protein *IAA12*, SCEQRT2093D08.g; and RNA polymerase II transcriptional coactivator *KELP*, SCCCLR2001H06.g) and down-regulated genes (a bZIP family member *TGAL3*, SCSGFL4194F09.g and nuclear transcription factor Y subunit A-10, SCRLLR1109E12.g). Most of these genes have already been reported in plant responses to abiotic stresses^29,41–43^. For example, *A. thaliana* transgenic lines overexpressing TaNF-Y10 were more sensitive to water and salt stress, as compared to wild-type plants^41^. Furthermore, overexpression of *TaMADS51* improves growth, biomass yield, and phosphate accumulation in tobacco plants, as well as the antioxidant enzymatic activities, specifically under phosphorus-starvation conditions^42^. Interestingly, a network-based study in sorghum revealed the responsiveness of MADS-box and bZIP transcription factors to co-occurring stresses, including drought^29^, which may cross-talk with some transcriptional responses of sugarcane found herein. Thus, our results indicate that the drought tolerance of the ‘IACSP94-2094’ genotype may be partially explained by an enhanced transcriptional regulation activity, which has conserved elements with sorghum drought-related pathways.

Carbohydrate and amino acid metabolism act synergistically in cellular acclimation to environmental stresses^44–46^. The enriched carbohydrate metabolism pathway comprises genes that are most significantly repressed in both genotypes under drought stress; except for trehalose-6-phosphate synthase (T6P, SCCCCL2001H04.b), which plays a role in osmoprotection and dramatically improves drought tolerance when overexpressed in model plants^47,48^. Interestingly, the tolerant genotype ‘IACSP94-2094’ exhibits a particular altered amino acid metabolism under severe drought stress, relying on up-regulated genes, such as NADH-dependent glutamate synthase 1 (GLT1, SCCCCL3120F01.g), glutamate decarboxylase 1 (GAD1, SCCCRZ2C03D05.g), and aspartate kinase-homoserine dehydrogenase (AK-HseDH, SCACCL6010C02.g). GLT1 and GAD1 genes are involved in glutamine synthetase/glutamate synthase (GOGAT/GS) cycle and the γ-Aminobutyric acid (GABA) metabolism, which are associated with the maintenance of the tricarboxylic acid (TCA) cycle and nitrogen assimilation in wild tomato (*Solanum pennellii*), serving as key players in drought tolerance^49^. These results suggest the metabolism of amino acid and nitrogen as an additional layer of the adaptive molecular machinery of ‘IACSP94-2094’ genotype.

Protein accumulation in leaves has been proposed as a beneficial process for plants subjected to drought^50^. Proteomic and metabolomic studies reported significantly increased abundances of ribosomal proteins (RPs) in sugarcane leaves under water-limiting conditions in greenhouse^33,51^. Accordingly, both sensitive and tolerant sugarcane genotypes showed a significantly enriched translation pathway under severe drought stress. Remarkably, 11 DEGs were particularly uncovered in ‘IACSP94-2094’, among which 10 correspond to up-regulated genes, such as 40S (RPS2a, SCCCLR1001D03.g; RPS7, SCVPLR2005E09.g; RPS11, SCCCCL4002F03.g; RPS18, SCCCLR1001H01.g; RPS29, SCQGLR1086G07.g) and 60S (RPL9, SCBFRZ2045D04.g; RPL13a-4, SCCCCL4004E02.g; RPL22-2, SCQGAM1045C11.g; RPL32, SCCCLR1C07F11.g; RPL37a, SCCCRZ2002C01.g) ribosomal proteins. On the other hand, only two 60S ribosomal proteins (RPL2, SCVPLR2027H04.g; RPL11, SCVPLR2019F01.g) were particularly up-regulated in ‘IACSP97-7065’. Although the interplay between translation processes and drought tolerance in plants is still poorly elucidated, our results point to a clear difference in ribosome-related gene regulation between sensitive and tolerant sugarcane genotypes, suggesting a prominent protein restoration capacity of ‘IACSP94-2094’.

Natural field conditions harbor multiple biotic and abiotic stresses that trigger overlapping molecular networks in plants to withstand limiting environments^52^. For instance, some of the DEGs of both sugarcane genotypes are strictly associated with pathogen resistance, such as receptor-like serine/threonine-protein kinase (ALE2, SCCCLR1079G06.g). Therefore, not every DEG found in this study can be readily anticipated to be related to drought. Moreover, we also detected around 5% of hypothetical drought-induced proteins in leaves of ‘IACSP94-2094’ under severe drought stress. Both findings require further investigations, using gene editing/knockdown and/or gene overexpression strategies, combined with structural bioinformatics (structural modeling and molecular docking) to identify precisely the functions of those proteins. To illustrate, a putative 32.7 kDa jasmonate-induced gene (SCJLLR1103A10.g)—currently known as dirigent-jacalin (*ShDJ*)—was exclusively up-regulated in ‘IACSP94-2094’ under severe drought. Further, it was overexpressed in transgenic rice lines and improved drought tolerance, plant biomass accumulation, and saccharification efficiency^53^.

Finally, this study revealed the gene expression profiles underlying the differential responses of contrasting sugarcane genotypes for drought tolerance under field conditions. After 117 days of the last rainfall, the tolerant genotype displayed significant changes in biological pathways associated with transcriptional regulation, oxidoreductase activity, amino acid metabolism, and translation process. Collectively, these molecular traits portray the molecular basis of ‘IACSP94-2094’ to cope with severe drought stress. For example, (i) the high oxidoreductase activity might protect PSII against oxidative stress, thus favoring photosynthetic activity even under water shortage^32^; (ii) the enhanced amino acid metabolism may regulate the balance of compatible osmolytes, signaling molecules, and secondary metabolites precursors, which are critical in abiotic stress responses^54^; and (iii) the remarkable transcriptional regulation activity may orchestrate the expression of multiple genes, including those associated with drought tolerance^55^. Thus, our study contributes substantially to the understanding of drought-induced response networks in sugarcane leaves at a trustworthy water-limiting scenario, as well as the discovery of new putative genes, which together may provide novel strategies for plant breeding programs.

## Methods

### Plant material and drought treatment

To investigate the effect of drought stress on gene expression in contrasting genotypes, we used two sugarcane (*Saccharum* spp.) genotypes, ‘IACSP97-7065’ (drought-sensitive) and ‘IACSP94-2984’ (drought-tolerant). These genotypes were developed and selected by the “Programa Cana” (Instituto Agronômico, Ribeirão Preto, Brazil), and they display differential growth and yield in drought-prone areas of Cerrado. The plants were grown and evaluated under irrigated (water supplied by linear sprinkler system) and non-irrigated field conditions at ‘Jalles Machado’ Sugar Mill (Goianésia, Brazil, 15° 13′ S; 48° 56′ W; 591 m a.s.l.) during the dry season, from April to September. Irrigated plants were supplied with water (60 mm) at 15 days intervals. Each plot was composed of 40 plants and arranged in four rows of 5 m each, with three replicates. Soil water potential (Ψ, kPa) was monitored by WATERMARK probes (model 200SS, Irrometer, Riverside, CA, USA) at 0.6 m depth. More details about water management are described elsewhere^3^. Leaf samples (the first fully expanded leaf with visible ligule, i.e., leaf +1) of first-cut plants were collected between 9:00 and 9:30 a.m. in irrigated (control) and non-irrigated (treatment) areas at 42 (May) and 117 (August) days after the last rainfall, when plants were facing mild (soil water potential of −60 kPa) and severe (soil water potential of −75 kPa) drought stress, respectively. At 42 and 117 days after the last rainfall, the plants were 6 and 9 months old, respectively. The plant material used in the present study was sourced from the IAC sugarcane germplasm bank (Instituto Agronômico, Centro de Cana, Ribeirão Preto, Brazil) of our breeding program, coordinated by Marcos Landell, Silvana Creste, and Daniel Nunes (Instituto Agronômico, Centro de Cana, Ribeirão Preto, Brazil), with no licensing requirements for the authors. Experimental research and the field study reported herein are in compliance with relevant institutional and national, guidelines and legislation.

### Physiological analyses

The physiological responses of ‘IACSP97-7065’ and ‘IACSP94-2984’ were assessed under irrigated (control) and non-irrigated (treatment) conditions at mild and severe drought stress stages. Chlorophyll fluorescence was measured with a fluorometer coupled to an infrared gas analyzer (model LI-6400XT, LI-COR, Lincoln, NE, USA) and some photochemical parameters were estimated: the maximum (*F*_v_/*F*_m_) and effective (Φ_PSII_) quantum efficiency of PSII, and the non-photochemical quenching (*NPQ*). While *F*_v_*/F*_m_ is based on the minimum (*F*_o_), maximum (*F*_*m*_) and variable (*F*_v_ = *F*_m_ − *F*_o_) fluorescence signals in dark-adapted leaf tissues, Φ_PSII_ is based on the steady-state (*F*_s_), maximum (*F*_*m*_*′*) and variable (Δ*F* = *F*_*m*_*′* − *F*_s_) fluorescence signals in light-adapted leaves, following the saturation pulse method (λ < 710 nm, *Q* ~ 12,000 µmol m^−2^ s^−1^, 0.8 s)^56^. Chlorophyll a and b contents were also indirectly measured with a chlorophyll-meter (clorofiLOG, Falker, Brazil), by calculating the Falker Chlorophyll Index (FCI), which represents the chlorophyll mass/leaf mass ratio. In addition, the CO_2_ assimilation (*P*_n_) was evaluated, as a gas exchange-associated parameter, by using an infrared gas analyzer (model LI-6400XT, LI-COR, Lincoln, NE, USA). These measurements were performed on sugarcane leaves (the first fully expanded leaf with visible ligule, known as leaf + 1) under photosynthetic photon flux density of 2000 µmol m^−2^ s^−1^) and air CO_2_ concentration of 385 ± 2 ppm, considering the natural variation of air temperature (34.7 ± 1.0 °C) and relative humidity (41 ± 2%). Measurements were taken between 09:00 and 11:00 am, when leaf temperature was 34.6 ± 1.0 °C. Technical and biological triplicates were subjected to the three-way analysis of variance (ANOVA), followed by Fisher’s least significant difference test, which was performed using GraphPad Prism version 8.0.0 for Windows, GraphPad Software, San Diego, California USA (www.graphpad.com).

### Microarray procedures and data analysis

Total RNA was extracted from 1 g of leaf tissue by the lithium chloride (LiCl) method and purified using the RNeasy kit (QIAGEN, Hilden, Germany) according to the manufacturer’s instructions. Isolated RNA was quantified using a Nanodrop 2000 (Thermo Fisher Scientific, Waltham, MA, USA) spectrophotometer, and its integrity was evaluated via 1.2% agarose electrophoresis. Then, RNA samples were treated with the DNase I RNase-free (Fermentas, Vilnius, Lithuania).

The oligo microarray (4 × 44 K) contained 14,552 Sugarcane Assembled Sequences (SAS) released from Sugarcane EST Project (SUCEST) database^57^. The complementary RNA (cRNA) synthesis, dye labeling, washing, and hybridization of the samples were performed according to the Two-Color Microarray-Based Gene Expression Analysis protocol. Hybridized microarrays were scanned using the Agilent Microarray Scanner (Agilent Technologies, Santa Clara, CA, USA). Thus, two biological and technical replicates were used for each microarray experiment. Microarray data sets can be found at the Gene Expression Omnibus (GEO) (series, record GSE187416) and at the SUCEST database (http://sucest-fun.org/). The statistical analyses were carried out using the HTself method^58^ to determine the differentially expressed genes (DEGs) in each experiment (confidence level set to 0.9). For each experimental condition, DEGs were determined when at least 70% of the spots showed similar and positively correlated patterns in the two technical replicates. Finally, the gene expression was calculated and normalized in log_2_Fold-change (FC). To confirm the reliability of the microarray data, the Pearson’s and Spearman’s rank correlation coefficients (*p*-value ≤ 0.05) were determined using the log_2_FC values obtained from the microarray and RT-qPCR analyses. Pearson’s and Spearman’s correlation analyses were performed using GraphPad Prism version 8.0.0 for Windows, GraphPad Software, San Diego, California USA (www.graphpad.com).

### Functional annotation

Identification of orthologues from vascular plants (Tracheophyta–Taxonomy ID: 58023) was conducted for each DEG by using the BlastX tool in Blast2GO software version 5.2^59,60^ (https://www.blast2go.com/) with an e-value ≤ 10^−5^ cut-off. PlantGSEA^61^ was used for functional enrichment encompassing Gene Ontology (GO) terms and Kyoto Encyclopedia of Genes and Genomes (KEGG)^62–64^ pathways for all DEGs of both genotypes under mild and severe drought stresses. Fisher’s exact test was used to calculate the significance (*p*-value) of each enriched term, and Benjamini-Yekutieli’s for multiple testing correction. Plant-related significant enriched terms were selected with an *p* ≤ 0.05 cut-off.

### Reverse transcription-quantitative PCR analysis

Total RNA was extracted from sugarcane leaves using the TRIzol Reagent (Invitrogen, Carlsbad, CA, USA) and treated with DNase I (Promega, Fitchburg, WI, USA), oligo dT (0.5 µg µL^−1^), and 0.5 mM dNTP mix. RNA concentration was measured using a NanoDrop 2000 spectrophotometer (Thermo Fisher Scientific, Waltham, MA, USA), and RNA integrity was checked in 1.2% agarose gel electrophoresis stained with ethidium bromide (1 µg µL^−1^). First-strand cDNA was synthesized from 1 µg of total RNA with the RevertAid Reverse Transcriptase kit (Thermo Fisher Scientific, Waltham, MA, USA), according to the manufacturer’s instructions. RT-qPCR reactions were performed on the Rotor-Gene Q (QIAGEN, Hilden, Germany). Briefly, a 10 µL reaction mixture consisted of 5 µL Platinum SYBR Green qPCR SuperMix-UDG (Thermo Fisher Scientific, Waltham, MA, USA), 1 µL of diluted cDNA (1:10) with 0.3 µM primers concentration. The thermal reaction settings were set to an initial temperature of 95 °C for 2 min, followed by 40 cycles of 95 °C for 15 s, 60 °C for 20 s, and 72 °C for 20 s.

We randomly selected 20 DEGs for the RT-qPCR assay to confirm the gene expression patterns. Each primer pair is described in Supplementary Table S4 online. Melting curve analysis between 72 and 95 °C was performed to confirm the specificity of the reaction. For each sample, three biological and technical replicates were used (*n* = 3). The reference genes *rpl35-4*, *rRNA1*, and *ubq1* (see Supplementary Table S4 online) were used to normalization of the relative expression values. Finally, relative expression levels (drought treatment against irrigated control) were calculated using the comparative C_t_ method^65^, followed by log_2_ normalization.

## Supplementary Information

Supplementary Information 1.

Supplementary Information 2.

Supplementary Information 3.

Supplementary Information 4.

Supplementary Information 5.

## Data Availability

The datasets generated and analyzed during the current study are available in the Gene Expression Omnibus repository (Accession code: GEO series record GSE187416), https://www.ncbi.nlm.nih.gov/geo/query/acc.cgi?acc=GSE187416.
