# Supplementary material for: Leaf transcriptome profiling of contrasting sugarcane genotypes for drought tolerance under field conditions
Source: Sci Rep. 2022 Jun 1;12:9153. doi: 10.1038/s41598-022-13158-5 (PMC9160059; doi:10.1038/s41598-022-13158-5)
Supplement: Supplementary file 1 — Supplementary Information 1. [file 41598_2022_13158_MOESM1_ESM.pdf]

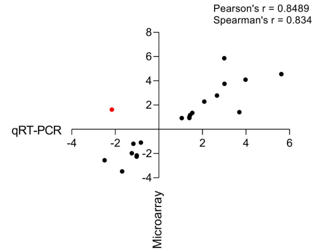

Supplementary Figure S1 – Correlation analysis between microarray transcripts and RT-qPCR expression data. Each dot corresponds to an experimentally validated transcript. The red dot corresponds to a gene with different expression between the microarray and RT-qPCR approaches.
